# Spatial Factors Play a Major Role as Determinants of Endemic Ground Beetle Beta Diversity of Madeira Island Laurisilva

**DOI:** 10.1371/journal.pone.0064591

**Published:** 2013-05-27

**Authors:** Mário Boieiro, José C. Carvalho, Pedro Cardoso, Carlos A. S. Aguiar, Carla Rego, Israel de Faria e Silva, Isabel R. Amorim, Fernando Pereira, Eduardo B. Azevedo, Paulo A. V. Borges, Artur R. M. Serrano

**Affiliations:** 1 Centro de Biologia Ambiental and Portuguese Platform for Enhancing Ecological Research & Sustainability, Faculdade de Ciências da Universidade de Lisboa, Lisboa, Portugal; 2 Azorean Biodiversity Group and Portuguese Platform for Enhancing Ecological Research & Sustainability, Departamento de Ciências Agrárias, Universidade dos Açores, Angra do Heroísmo, Açores, Portugal; 3 Finnish Museum of Natural History, University of Helsinki, Helsinki, Finland; 4 Centro de Estudos do Clima, Meteorologia e Mudanças Globais, Departamento de Ciências Agrárias, Universidade dos Açores, Angra do Heroísmo, Açores, Portugal; Roehampton University, United Kingdom

## Abstract

The development in recent years of new beta diversity analytical approaches highlighted valuable information on the different processes structuring ecological communities. A crucial development for the understanding of beta diversity patterns was also its differentiation in two components: species turnover and richness differences. In this study, we evaluate beta diversity patterns of ground beetles from 26 sites in Madeira Island distributed throughout Laurisilva – a relict forest restricted to the Macaronesian archipelagos. We assess how the two components of ground beetle beta diversity (β_repl_ – species turnover and β_rich -_ species richness differences) relate with differences in climate, geography, landscape composition matrix, woody plant species richness and soil characteristics and the relative importance of the effects of these variables at different spatial scales. We sampled 1025 specimens from 31 species, most of which are endemic to Madeira Island. A spatially explicit analysis was used to evaluate the contribution of pure environmental, pure spatial and environmental spatially structured effects on variation in ground beetle species richness and composition. Variation partitioning showed that 31.9% of species turnover (β_repl_) and 40.7% of species richness variation (β_rich_) could be explained by the environmental and spatial variables. However, different environmental variables controlled the two types of beta diversity: β_repl_ was influenced by climate, disturbance and soil organic matter content whilst β_rich_ was controlled by altitude and slope. Furthermore, spatial variables, represented through Moran’s eigenvector maps, played a significant role in explaining both β_repl_ and β_rich_, suggesting that both dispersal ability and Madeira Island complex orography are crucial for the understanding of beta diversity patterns in this group of beetles.

## Introduction

The variation in species richness and composition across space and/or time has long been a central issue in biogeography and macroecology. During the last decade this subject became a key research area where major efforts have been addressed to measure the degree of biological communities’ distinctness, to understand the underlying causes of compositional heterogeneity and to clarify the concepts and the methods used in this kind of studies [1]–[6]. The concept of beta diversity, originally defined as “The extent of change of community composition, or degree of community differentiation, in relation to a complex-gradient of environment, or a pattern of environments” [7], has been used to refer to a variety of phenomena that somehow translate the compositional heterogeneity of species assemblages among places [4]. Recently, Anderson et al. [6] provided a framework for the analysis of beta diversity taking in consideration a set of mission statements and proposed that two types of beta diversity should be considered - the change in community structure along a given gradient (turnover) and the variation in community structure among sampling units within a given area without reference to a particular gradient or direction. Independently of the perspective, beta diversity may be conceptualized as the result of two basic processes: i) the replacement of species, and ii) species richness differences among assemblages [8]–[14]. Therefore, it is useful to assess the relative role of each component in generating beta diversity and evaluate how different factors and scales determine their patterns. A number of ecological, evolutionary and historical factors are known to be determinant for species richness patterns and differences in species richness and composition between habitats. For instance, Nekola & White [15] identified two main causes for the increasing dissimilarity among assemblages along geographic distance. According to these authors, the decrease in environmental similarity with distance may be due to competitive species sorting as a result of interspecific differences in physiological/ecological requisites (the niche difference model). Furthermore, they argued that the decrease in similarity of species assemblages along geographic distances may translate the dispersal of organisms across landscapes, a process strongly influenced by the spatial context and configuration of habitats together with time (the model of temporal and spatial constraint). In the last few years many studies have focused in disentangling the relative importance of environmental and spatial factors in explaining the differences in species richness and composition between sites and they have also emphasized that the role played by each factor in structuring communities is strongly associated with the scale of analysis [16]–[20]. Spatial scale dependency of the factors driving beta diversity has been clearly outlined in recent studies for a variety of ecological and taxonomic groups (e.g. [20]–[22]).

Oceanic island ecosystems have proved to be very useful for the understanding of the role of different phenomena in shaping community assembly [23]. These ecosystems are, in general, biologically simpler than mainland counterparts, well-defined geographically and usually their history can be traced back to their origin. As such, comprehensive studies on island biodiversity have helped to clarify the role of ecological and evolutionary phenomena in shaping spatial patterns of species richness and composition (e.g. [24]–[27]). The Macaronesian archipelagos harbour a unique type of laurel forest, the Laurisilva, which is a relic of the subtropical forests that covered the west Mediterranean area during the Tertiary [28]. Madeira Island comprises the most pristine and largest continuous area of Laurisilva with a large number of endemic species associated, particularly insects [29], [30]. Beta diversity studies on forest insects are still scarce even for some emblematic and well-studied groups and the information on drivers of variation in species richness and composition is not concordant. Several studies have emphasized the role of vegetation structure and composition as strong determinants of insect assemblages worldwide, since plants act as the physical habitat for many insect species and many others are intimately associated with specific plant species [31]–[33]. However, other studies found that changes in elevation were a better predictor of insect species turnover than variables related with vegetation composition and structure [34]–[35]. These studies showed that in spite of the significant correlations between vegetation diversity, composition and structure with species richness and composition of insect assemblages, those relationships were in fact mediated via environmental variables (e.g. temperature, humidity, altitude).

In this study we examine the relative roles of species replacement and species richness differences in generating beta diversity patterns of ground beetles occurring in a relic forest, the Madeira Laurisilva. We also assess the effects of environmental and spatial variables on the two components of beta diversity and the effect of spatial scale on the relative importance of those variables on beta diversity patterns.

## Materials and Methods

### Ethics Statement

Permission to conduct fieldwork in Laurisilva was obtained from the Madeira Natural Park and two nature wardens accompanied the study. Field studies did not involve endangered or protected species and were carried out in accordance with national and international laws.

### Study Area

Our study took place in Madeira, an oceanic island located 600 Km off the Atlantic coast of North Africa, with an area of 742 km^2^ and a maximum altitude of 1861 m (at Pico Ruivo). Madeira Island dates back to the late Miocene (∼5.6 My) with the most recent volcanic activity recorded 6,000–7,000 years ago. One distinctive feature of Madeira landscape is the presence of a relic subtropical forest - the Laurisilva - a vegetation type that originally covered much of the Mediterranean Basin before climate became much drier and harsher. The Laurisilva is a forest-type dominated by sclerophyllous laurel tree species from the genera *Apollonias*, *Laurus*, *Ocotea* and *Persea*, together with *Clethra arborea*, *Ilex perado* and *Morella faya* [28], [36], [37]. In Madeira, the Laurisilva is located mainly in the northern part of the island usually between 300 and 1400 meters altitude, with the most pristine fragments being generally found in areas of difficult access [28], [36]. Madeiran orography played a key role on the survival of Laurisilva by hindering human settlement in many areas of the island. Aware of the natural legacy of the island, local authorities created in 1981 the Natural Park of Madeira, a protected area covering about two-thirds of Madeira Island and including almost the whole area of Laurisilva. More recently, due to its outstanding natural value, Madeira Laurisilva was included in the World Heritage List [38] and the same reason has led to the inclusion of Madeira archipelago in the Mediterranean biodiversity hotspot [39], [40].

This study sampled 26 sites distributed throughout Madeira Laurisilva (see details on sampling site locations in Appendix S1). Site selection was constrained by the possibility of applying the sampling protocol and aimed at sampling as extensively as possible and to include isolated forest fragments. Furthermore, site selection was restricted mostly to pristine or near pristine forest fragments. The dominant tree species in the study areas were the Macaronesian endemics *Clethra arborea*, *Erica platycodon maderincola*, *Laurus novocanariensis*, *Ocotea foetens* and *Persea indica*, and the native *Morella faya*. A more detailed description of the geography and floristic composition of the forest fragments where sampling took place can be found in Neves et al. [36].

### Ground Beetle Sampling

Ground beetles (Coleoptera, Carabidae) are a charismatic beetle group frequently used as bioindicators in biodiversity and conservation studies [41], [42]. In spite of the large amount of literature on European forest carabids, there is still a gap on the knowledge on the assemblages associated with Laurisilva. We sampled ground beetles by applying a standardized sampling protocol developed for epigean invertebrates. This methodology proved to be both efficient and effective in sampling ground beetles in native laurel forest fragments in the Azorean islands, where it has been repeatedly used in inventorying and monitoring programs (e.g. [25], [43], [44]). In each site a linear transect of 30 pitfall traps (plastic cups with 4.2 cm diameter and 7.8 cm height) spaced between them by 5 meters was set. Pitfall traps were filled either with ethylene glycol (10%) or Turquin solution [43], together with some drops of detergent to reduce surface tension, and were then set along a linear transect with the two solutions disposed in an alternate way. The traps were covered with a plastic cover (15 cm diameter) fixed 3–4 cm aboveground to prevent flooding and loss of specimens due to heavy rain. The sampling took place during May-June, a period when a high number of ground beetle species is active, during two consecutive years: sites 1–20 were sampled in 2006 and sites 21–26 in 2007 (Appendix S1). Traps were active in the field for a two-week period and then the samples were brought to the lab, where the specimens were sorted and identified to species level. All specimens were deposited in the entomological collection of the Animal Biology Department (Faculty of Sciences, University of Lisbon, Portugal). Accidental captures of ground beetle species that have arboreal habits were not considered for the analysis.

### Partitioning Beta Diversity

To quantify the variation in species composition between sites we performed a beta diversity partitioning analysis [12]–[14]. For a pairwise comparison of sites, the total number of species may be decomposed into three quantities: the number of species common to both sites (a) and the number of species unique to each site, (b) and (c). From this, we may calculate absolute beta diversity (b+c) and evaluate the relative roles of replacement (2 min(b,c)) and difference in species richness (|b-c|) in generating beta diversity patterns. By scaling these quantities in relation to the total number of species in the system (a+b+c), which is theoretically linked to the notion of gamma diversity, we obtain the following equation:
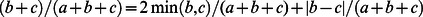



This algebraic decomposition may be summarized in the formula:
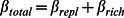
where, β_total_ represents the total community variation, β_repl_ gives the variation due to species replacement and β_rich_ accounts for the variation due to species richness differences (see a schematic representation of these equations in Carvalho et al. [14]).

### Environmental Variables

A number of environmental variables were collected during fieldwork to characterize sampling sites and such information was complemented by accessing a 100 m resolution GIS database for Madeira Island. In each study site we recorded the depth of forest litter (Litter), the number of dominant tree species (NumSpp) and we collected soil samples for pH (Soil_pH) and organic matter content (OrgMat) analyses. These variables were selected because they proved to play a role on the distribution and activity of forest ground dwelling arthropods in previous studies (e.g. [45]–[48]). Furthermore, the following climatic and physiographic variables were obtained from the GIS database for Madeira: nearest distance to urban areas (DUrb), altitude (Altitude), average slope (Slope), annual precipitation, minimum and maximum relative humidity, and minimum and maximum annual temperature. Madeira Island local climate data was obtained from a model developed in a GIS environment following the methodology proposed by Azevedo [49] for island ecosystems. Due to the high correlation among the climatic variables, we combined them into two factors by principal component analysis (PCA). From the PCA, we extracted the first two principal component eigenvectors (PC1_climate and PC2_climate) that together explained 94.6% of the variability in climate data. The first factor (PC1_climate) explained 74.2% of the variability in climatic data (eigenvalue = 3.7123) and represented a gradient of increasing temperature (negative scores) against an increasing gradient of relative humidity (positive scores). The second factor (PC2_climate) explained 20.4% of the variability in climatic data (eigenvalue = 1.0186) and represented a gradient of increasing precipitation (positive scores) against higher values of temperature and relative humidity (negative scores).

A landscape disturbance variable (Disturb) was created following a recently proposed metric based on the attributes of landscape matrix [50]. This variable corresponds to a local index of disturbance by taking into account the level of disturbance in the surrounding areas. It was calculated, in a first instance, by attributing a score to each habitat type found in Madeira and taking in consideration the increasing level of disturbance detected among them. The scores were defined as follows: Natural forests = 0; Natural(ized) vegetation/Rocky areas = 1; Exotic forest = 2; Pastures = 3; Orchards = 4; Urban/Industrial = 5. Then, the landscape disturbance index of each 100×100 m grid cell was calculated using the following formula:
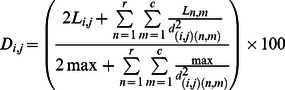
where:

D = landscape disturbance of the cell;

L = local disturbance of each cell;

r = number of rows in the map;

c = number of columns in the map;

d = distance between two cells;

max = maximum theoretical value of disturbance each cell may take (5 in the present case).

It is important to emphasize that the landscape disturbance index accounts for distance-dependent effects of disturbance, double weights the value of the focal cell compared with the adjacent ones and the values obtained fit in a 0 (no disturbance) to 100 (maximum disturbance) scale. Prior to the analysis, Disturb was log-transformed to improve normality. All variables were standardized to 0 mean and 1 SD to avoid scale effects. The final variables selected for the environmental matrix were: Litter, NumSpp, Soil_pH, OrgMat, DUrb, Altitude, Slope, Climate_PC1, Climate_PC2 and Disturb (Table 1).

**Table 1:** Average (±S.D.) values of the selected environmental variables from the 26 study sites.

Variable	Average±S.D.
Litter	3.6±1.2 cm
NumSpp	3.7±0.8
Soil_pH	5.4±0.4
OrgMat	36.7±13.4%
Durb	2167.5±795.1 m
Altitude	871.0±197.3 m
Slope	21.8±13.6°
Precipitation	1779.7±547.6 mm
Relative humidity (maximum)	97.5±2.0%
Relative humidity (minimum)	88.3±7.1%
Temperature (maximum)	15.9±2.0°
Temperature (minimum)	10.4±2.0°
Disturb	25.3±3.4%

Litter, litter depth; NumSpp, number of dominant tree species; OrgMat, percentage of organic matter content in soil; DUrb, nearest distance to urban areas; Disturb, percentage of landscape disturbance.

### Spatial Variables

The spatial variation in our dataset was modeled using Moran’s Eigenvector Maps (MEM), following a data driven approach described in Dray et al. [51]. Basically, MEM allows obtaining a set of spatial descriptors (eigenvectors) from site coordinates, a network describing the connection between sites and a weighting scheme for the connections. We examined four ways of defining neighbour networks, corresponding to a hierarchy of increasing connectivity: minimum spanning tree, relative neighbourhood graph, Gabriel graph and Delaunay triangulation [52]. In order to weight the connections between sites we tested two different schemes: i) binary weights, in this case no spatial weights are required, sites are considered connected (1) or not (0); and ii) weighting functions based on Euclidean distances among sites, assuming that a process influences local assemblages with an intensity decreasing with distance. Two weighting functions were tested: *f1 = 1/d* and *f2 = 1−d/d_max_* where *d* represents the Euclidean distance between sites and *d_max_* the maximum distance between two connected sites. In total we obtained 12 different spatial models. The positive eigenvectors of each model represent positive spatial correlation and were retained as explanatory spatial variables in subsequent analysis. All the procedures were carried out using the package spacemakeR [53] for the R language [54].

### Data Analysis

We performed an analysis of biotic dissimilarity with geographic distance, which is the complement of distance-decay of similarity [15], by regressing the pairwise dissimilarity β_total_, β_repl_ and β_rich_ matrices against the Euclidean distances between sites. A Mantel test was then applied to assess the significance of the relationship between biotic dissimilarity and geographic distance [55]. Significance tests were performed by permutation (1000 permutations).

For the construction of the environmental and spatial models, the β_repl_ and β_rich_ dissimilarity matrices were regressed against environmental and spatial explanatory variables, using Canonical Analysis of Principal Coordinates (CAP). In order to construct the environmental model, we ran a forward selection procedure, using CAP, on the environmental dataset to select those variables with a significant contribution (P<0.1 after 9999 random permutations) to explain variation in β_repl_ and β_rich_ dissimilarity matrices. We followed the recommendations proposed by Blanchet et al. [56] to avoid inflated Type I error and the overestimation of the amount of explained variance.

For modeling the spatial structure of β_repl_ and β_rich_ we used the procedure described in Dray et al. [51] to select the most parsimonious model from the initial set of 12 candidates. Briefly, we: i) calculated the positive MEM eigenvectors, representing positive spatial correlation; ii) used CAP scores to regress β_repl_ and β_rich_ against the MEM eigenvectors; iii) reordered the MEM eigenvectors according to their explanatory power; iv) entered MEM eigenvectors one by one into the model; and v) retained only the MEM eigenvectors that correspond to the regression model with the lowest corrected Akaike Information Criterion (AIC_c_). When this procedure was done for all candidates, we retained the most parsimonious spatial model (lowest AIC_c_). Finally, we used variation partitioning to quantify the proportion of the variation in species replacement (β_repl_) and richness differences (β_rich_) explained by purely environmental, purely spatial and spatially structured environmental effects [57]. Partitioning was carried out through a series of partial CAP, using adjusted R^2^ values as suggested by Peres-Neto et al. [58]. The mantel tests, CAP and variation partitioning procedures were performed in the R statistical language [54] using the package ‘vegan’ [59].

## Results

A total of 1025 individuals belonging to 31 ground beetle species were collected in this study. Endemic species were clearly dominant in all the assemblages, with a total of 25 species recorded, while native non-endemic and introduced species were found in much lower numbers, 4 and 2, respectively (see Appendix S2). The mean observed species richness per site was 4.9 (SD = 1.5), with a minimum of 3 species and a maximum of 8 species recorded in the 26 study sites.

**Figure 1:**
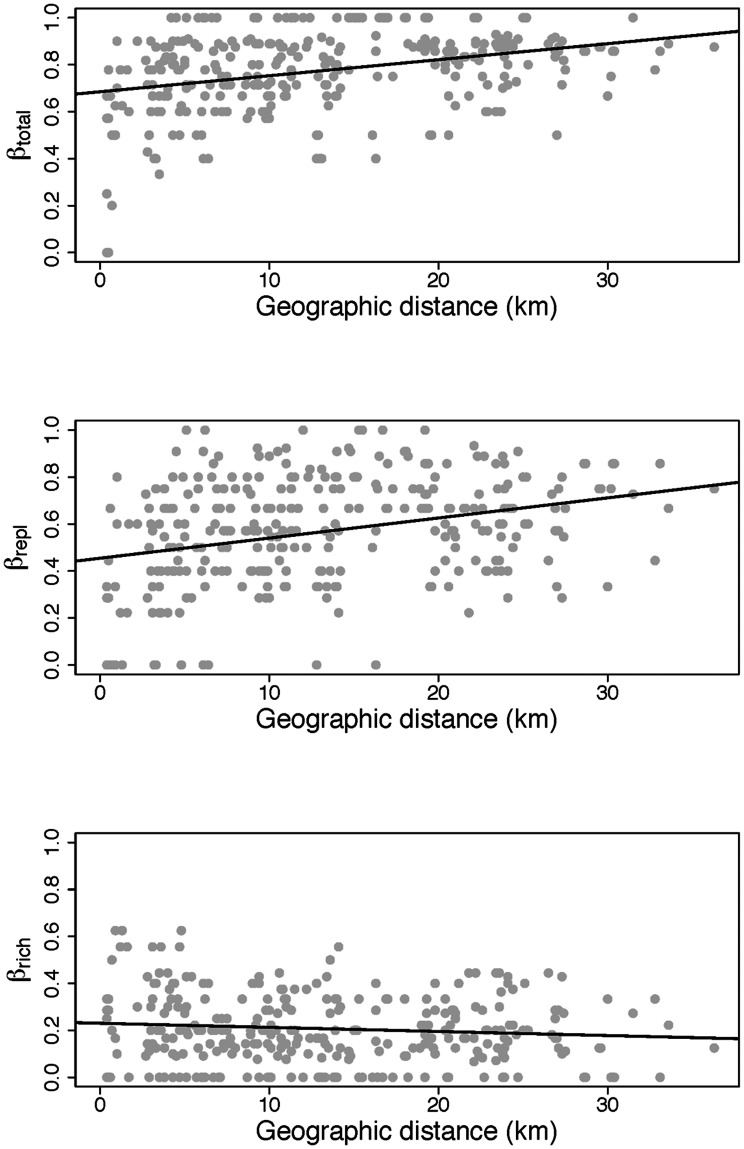
Distance decay in ground beetle assemblages’ dissimilarity. Distance dissimilarity plots representing the relationship between ground beetle assemblage dissimilarity and geographic distance. β_total_, β_repl_ and β_rich_ were used as measures of overall beta diversity, beta diversity due to species replacement and beta diversity due to richness differences, respectively.

Mean pairwise dissimilarity was 0.779 (SD = 0.170) for the β_total_ matrix, 0.573 (SD = 0.230) for the β_repl_ matrix and 0.206 (SD = 0.146) for the β_rich_ matrix. Moreover, overall dissimilarity (β_total_) increased with geographic distance (intercept = 0.6848; slope = 0.0068; Mantel r = 0.3489; p = 0.001). However, by disentangling β_total_ in its two components, species replacement (β_repl_) and richness differences (β_rich_), we find contrasting results (Fig. 1). β_repl_ increases with geographic distance (intercept = 0.4545; slope = 0.0086; Mantel r = 0.3244; p = 0.001), whilst β_rich_ decreases (intercept = 0.2303; slope = −0.0017; Mantel r = −0.1039; p = 0.03).

**Figure 2:**
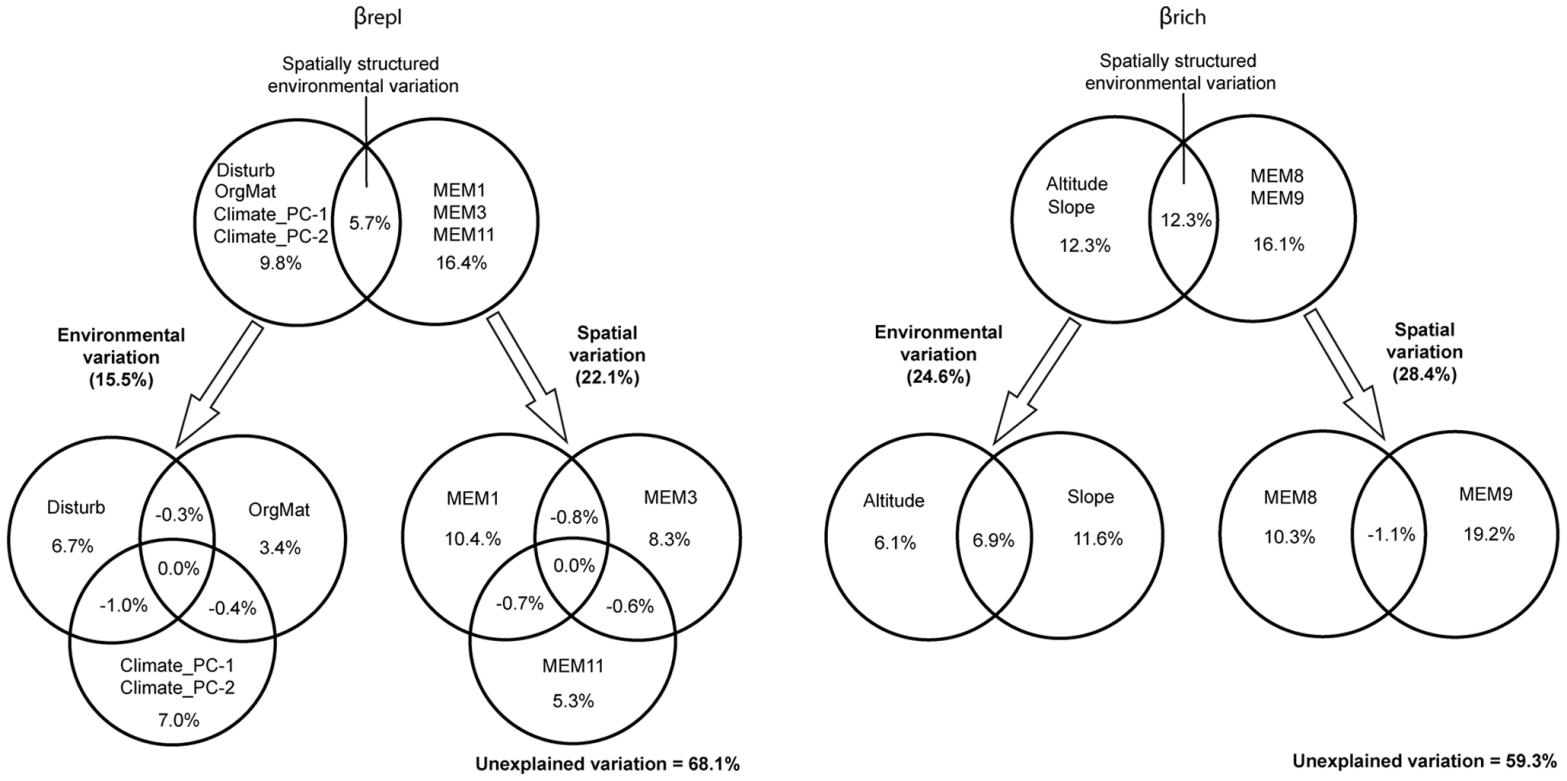
Variation of β_repl_ and β_rich_ dissimilarity explained by environmental and spatial variables and their shared effects. Venn diagrams showing the results of the variation partitioning procedure carried out on the forward selected environmental and spatial (Moran’s eigenvector maps) variables for both components of beta diversity: β_repl_ and β_rich_.

**Figure 3:**
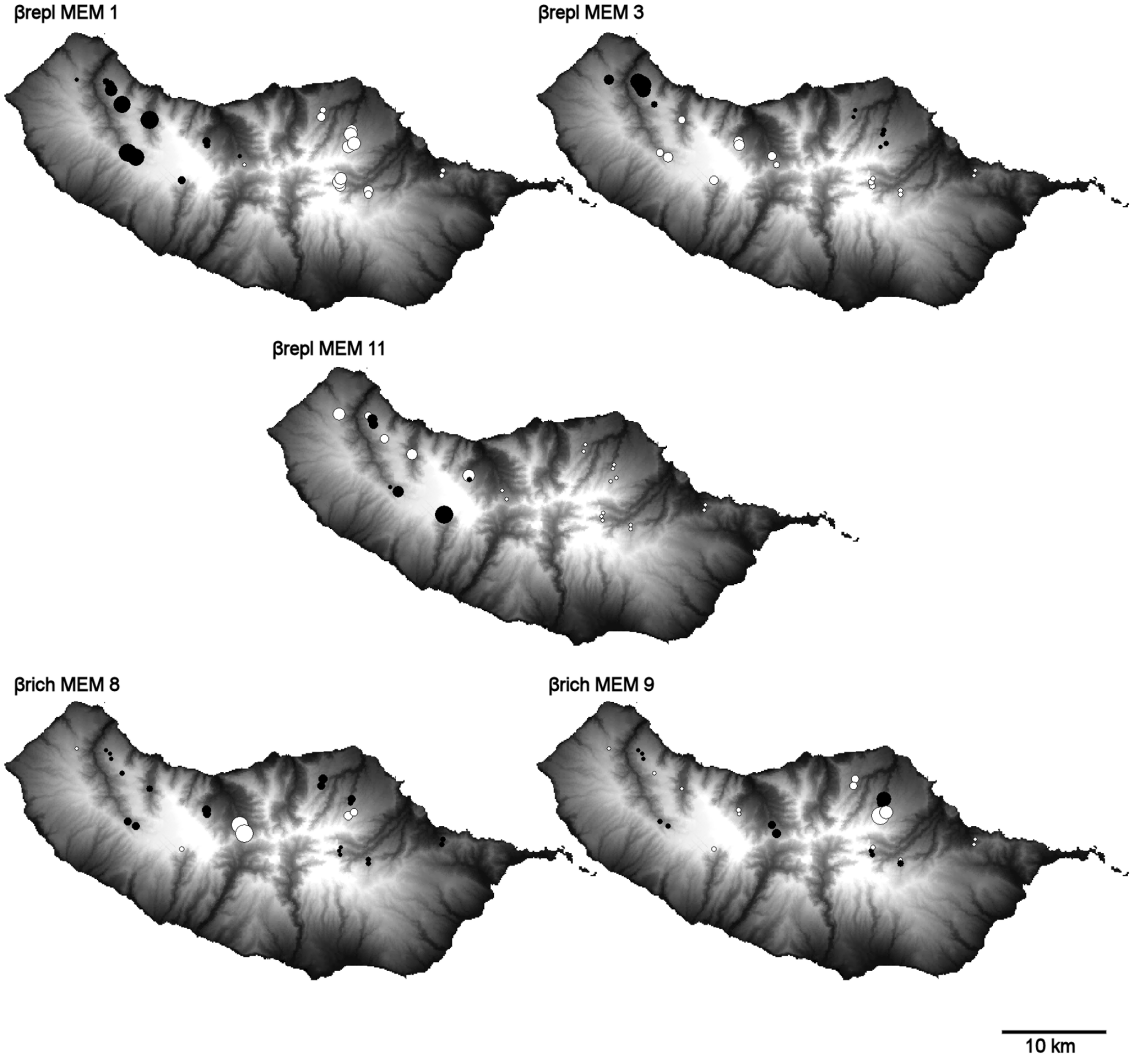
Geographic structure of differences in species richness (β_rich)_ and composition (β_repl)_ among assemblages by MEM variables at different spatial scales. Spatial representation of the selected MEM variables used to define the spatial models for the dissimilarity matrices β_repl_ (MEMs 1, 3 and 11) and β_rich_ (MEMs 8, 9). Each circle represents the position of a local assemblage (in a geographic coordinate system). For the selected MEM variables, white circles represent negative scores and black circles represent positive scores. Circle size is proportional to the absolute value of the site scores. MEM variables represent broad (a, b) and fine (c, d, e) spatial scales.

Considering the influence of environmental variation over the species replacement component, the forward selection procedure selected four environmental variables: Disturb, OrgMat, climate_PC1 and climate_PC2. This model explained 15.5% of the variation in β_repl_, being climatic effects (climate_PC1 and climate_PC2) and the landscape disturbance index more important than organic matter (Fig. 2). To explain the spatial variation of β_repl_ the most parsimonious model was the one originating from a Gabriel connectivity network of sites, where links were weighted by the *f2* function. Three Moran’s eigenvectors (MEM 1, 3 and 11) were retained as spatial descriptors of spatial pattern of β_repl_ and explained 22.1% of its variation (Fig. 2). MEM 1 and 3 represented broad scale variation, whilst MEM 11 represented fine scale spatial structure (Fig. 3). From the three spatial structures, MEM 1 had the highest contribution to explain β_repl_ (10.4% of variation), representing the differentiation between western and eastern assemblages. Variation partitioning revealed that 31.9% of the variation of the β_repl_ can be explained by the environmental and the MEM variables in conjunction (Fig. 2). The pure spatial component explained 16.4% of the variation, while 15.5% of the variation corresponds to environmental control (pure environmental = 9.8%; spatially structured environmental variation = 5.7%).

Regarding the environmental effects over the species richness differences component, the forward selection procedure selected two variables: Altitude and Slope. This model explains 24.6% of the variation in β_rich_, being Slope more important than Altitude (Fig. 2). To explain the spatial variation of β_rich_ the most parsimonious model was the one originating from a Delauney connectivity network of sites, where links were weighted by the *f1* function. Two Moran’s eigenvectors were retained as spatial descriptors of spatial pattern of β_rich_ (MEM 8 and 9) explaining 28.4% of its variation. These eigenvectors model fine scale structures (Fig. 3). Variation partitioning revealed that 40.7% of the variation of β_rich_ can be explained by the environmental and spatial variables in conjunction. The pure spatial fraction corresponds to 16.1% while environmental control explains 24.6% (pure environmental = 12.3%; spatially structured environmental variation = 12.3%) of the variation in β_rich_ (Fig. 2).

## Discussion

Madeira Island Laurisilva, as a whole, is still in a pristine condition, as revealed by the large proportion of endemic ground beetles found in this study. In fact, 29 out of the 31 species recorded were native or endemic to Madeira with the remaining two species being considered introduced [29] and occurring in low abundance and frequency in Laurisilva. The local species richness was within the range expected for this habitat-type based on previous studies where the same sampling technique was applied [60], [61]. These values show striking differences from those recorded in the Laurisilva fragments from the Azorean islands, where a much lower number of species and endemics were found [44]. The poor diversity in the Azores has been explained by island geological age, distance from the nearest source of propagules, geomorphology, forest structure and composition, and habitat disturbance [24], [44], [62].

A main finding of this study is that species replacement is more important than differences in species richness in structuring carabid beta diversity patterns. The low difference in species richness between sites may be a consequence of selecting sampling sites exclusively in pristine or near pristine forest patches, which presented similar woody vegetation structure and composition. Furthermore, the study took place in a relatively small range, with the largest distance between study sites being around 30 km. Thus, the combination of these sampling particularities may have contributed to target quite similar Laurisilva plant communities where large differences in carabid species richness are not expected to occur. A large number of studies on beta diversity, however, have focused on comparisons at much larger scales, encompassing a variety of habitat-types, ecoregions or even biomes, where large differences in species richness are known to occur [9], [63]–[65]. For example, a recent study using also ground beetles as model organisms showed a clear latitudinal gradient of species richness across Europe, with larger southern countries having a much higher number of species than northern ones [66].

A significant positive trend of species composition dissimilarity with geographic distance was found for Madeira carabids with assemblages from closer sites showing higher affinities in species composition. The distance decay of similarity in ecological assemblages is well documented, but controversy still remains on the relative importance of its drivers and on how it varies across organisms and environments [67], [68]. Furthermore, information on species traits, trophic level and dispersal capability proved to be important factors for the interpretation of distance decay patterns (e.g. [69]). The turnover among assemblages of Madeira Laurisilva ground beetles is jointly explained by pure environmental predictors, pure spatial variables and spatially structured environmental variation. Climatic variables together with soil organic matter content and landscape disturbance seem to play a role in ground beetle species replacement among forest patches. Climate variables have been frequently considered good predictors of variation in the composition of assemblages of diverse taxonomic groups since species differ on their performance under different environmental conditions and this may lead to species sorting according to spatial differences in climate [20], [70], [71]. The complex orography of Madeira Island plays a role on the genesis of the mosaic of local climatic conditions, which in turn provide a variety of conditions to living organisms leading to species sorting according to their ecological and physiological tolerances. A number of studies have shown the importance of climatic variables (e.g. temperature, relative humidity) in explaining ground beetle species’ distribution at different spatial scales and the occurrence of species segregation even at the microspatial level [44], [66], [72]–[74]. Other environmental variables, such as plant species composition, litter depth, soil pH and organic matter content are also known to be important in determining local species composition of forest ground beetles (e.g. [72], [73]). Among these, only differences in soil organic matter content among sampling sites seemed to play a role in explaining variation in ground beetle assemblages’ composition in our study. This may be an indirect effect of prey availability since carabid prey (earthworms, snails, springtails, etc.) are intimately associated with soil properties. Thus, changes in soil characteristics may lead to differences in prey abundance and composition that can translate into compositional and structural changes on ground beetle assemblages as shown in several other studies [75], [76]. Habitat matrix composition also influenced variation in ground beetle assemblages. Habitat fragmentation and isolation together with land use changes of surrounding habitats are known to influence forest beetle assemblages by leading to differentiation between patches due to species loss and turnover with non-typical forest species (e.g. [77], [78]). In Madeira Island, many Laurisilva patches are still in a pristine condition but small and isolated fragments are more prone to species invasion from surrounding habitats. This is clearly illustrated by the case of the Rabaças fragment, where non-typical forest species – *Harpalus attenuatus*, *Ocys harpaloides* and *Microlestes* spp. – were recorded in small numbers together with typical forest species. In recent years, a number of studies have highlighted the important role played by matrix habitat on the ecological dynamics of heterogeneous landscapes (see a review by [79]), including some reports using ground beetles as model organisms [80], [81]. Although we acknowledge that our findings need a thorough support from studies specifically designed to account for the effects of landscape disturbance on ground beetle assemblages, we reinforce the need for the inclusion of those effects in ecological models if we aim to better understand the distribution and abundance of organisms.

Species richness differences among sites were mainly explained by Slope and Altitude, emphasizing the crucial role played by island geomorphology in structuring spatial patterns of diversity. These two variables influenced carabid assemblages in a direct way, but also indirectly by determining local environmental conditions. The upper limit of the Laurisilva had a slightly higher number of species than lower and intermediate altitudes since several ground beetle species were only found in locations above 900 m (e.g. *Bradycellus* spp., *Calathus complanatus*, *Cymindis maderae* and *Orthomus dilaticollis*). Several of these species can be found both in Laurisilva as well as in the transition of forest to altitudinal meadow suggesting that the increase in species richness at higher altitudes can result from the encounter of ground beetle assemblages with different environmental adaptations.

Pure spatial effects were found to play an important role in determining both species replacement and richness differences among sites. The spatial component may represent the signature of neutral and niche processes (e.g. dispersal limitation and biotic interactions) and also the effects of some unmeasured environmental variables that are spatially structured [2], [82]. Most Madeira Island endemic ground beetle species are wingless or lack functional wings suggesting that dispersal limitation may play a key role in community structure, particularly if we take in consideration the complex orographic environment where these species occur. This fact, together with the historical dynamics of Madeira, that included repeated volcanic events, slumping episodes and climate and sea-level changes (e.g. [83]), are probably the main drivers of species differentiation in this island and may help us understand the restricted distribution of some endemic ground beetles, particularly within genera that radiated in Madeira Island (e.g. *Orthomus* and *Trechus*) [30]. For instance, a distinctive feature of Madeira landscape that seems to have favored the differentiation of ground beetle communities in terms of species composition is the presence of deep valleys crossing the island from North to South that limited gene flow in organisms with low dispersal ability (Fig. 3). The geological, geographic and climatic historical dynamics of Madeira has been a key factor for the understanding of the high genetic and taxonomic diversity of the biota of this island and the observed high species turnover in different taxa [29], [84]–[87]. For example, Cook [87] highlighted that Madeiran land snail diversity is, to a large extent, due to the fortuitous coincidence of rates of geological and climatic change and geographic isolation, which have produced a number of isolates where species differentiation took place. Historical processes are known to influence the spatial configuration of species assemblages and their effects seem to be most important for taxonomic groups with poor dispersal capabilities [88]–[91]. In fact, a number of studies came to the conclusion that the combined effects of dispersal limitation and historical processes may hamper many narrowly-ranged species in reaching equilibrium with their environmental niche [92]–[94].

In Madeira Laurisilva, spatial processes played an important role as determinants of ground beetle beta diversity, but operated at different scales in structuring communities by species turnover and species richness differences (Fig. 3). Differences in species composition were mainly determined by pure spatial effects operating at larger scales. For instance, the most important spatial structure in explaining species replacement was MEM 1, which corresponds to the differentiation of western and eastern side assemblages of Madeira, separated by deep valleys with N-S orientation. Whereas, differences in species richness between sites seem to be determined by pure spatial effects and spatially structured environmental variation (related with topography) acting at finer scales (local variation). This finding reinforces the conclusions from other studies where beta diversity patterns were found to be the result of different processes, operating at different scales in the two distinct components of beta diversity [13], [14], [95], [96]. Overall, much of the observed variation in Madeira Laurisilva ground beetle communities remained unexplained. This may be due to methodological limitations of our study as well as to the so-called stochastic mechanisms and the effects of unmeasured environmental factors (e.g. microclimatic variables, structural habitat factors, abundance of competitors and predators). For instance, Judas et al. [97] have shown that several microclimatic variables may scale up to distributions at the landscape level for some ground beetle species. In conclusion, our findings suggest that beta diversity of Madeira Laurisilva ground beetles is mostly determined by changes in species composition (not richness) among sampling units. Spatial factors have a major role in explaining species replacement and variation in species richness between forest sites, suggesting that dispersal limitation and historical events are determinant factors in shaping patterns of biodiversity for this group of beetles. Furthermore, this study illustrates that a clearer interpretation of the role of drivers of beta diversity can only be achieved within a multi-scale framework where both components of beta diversity – species turnover and richness differences – are jointly assessed.

## Supporting Information

Appendix S1 **Detailed sampling site locations.** List of sampling sites names and their geographic coordinates (in decimal degrees) and altitude (in meters).
(DOC)

Appendix S2 **List of ground beetle species.** List of ground beetle species found in 26 sites in Madeira Island Laurisilva, including information on their distribution status.
(DOC)
