# Supplementary material for: Spatial Factors Play a Major Role as Determinants of Endemic Ground Beetle Beta Diversity of Madeira Island Laurisilva
Source: PLoS One. 2013 May 27;8(5):e64591. doi: 10.1371/journal.pone.0064591 (PMC3664619; doi:10.1371/journal.pone.0064591)
Supplement: Appendix S1 — Detailed sampling site locations. List of sampling sites names and their geographic coordinates (in decimal degrees) and altitude (in meters). (DOC) [file pone.0064591.s001.doc]

Appendix S1 – Site code, location, geographic coordinates (in decimal degrees) and altitude (in meters) of the sampling sites.

| **Site code** | **Location** | **Latitude** | **Longitude** | **Altitude** |
| --- | --- | --- | --- | --- |
|  |  |  |  |  |
| **1** | Funduras | 32.7493 | -16.8114 | 500 |
| **2** | Funduras | 32.7540 | -16.8099 | 552 |
| **3** | Fajã da Nogueira – Lvda. Pte. Roquete | 32.7391 | -16.9156 | 1074 |
| **4** | Fajã da Nogueira – Tanque | 32.7425 | -16.9168 | 845 |
| **5** | Fajã da Nogueira - Til Gigante | 32.7457 | -16.9150 | 841 |
| **6** | Queimadas | 32.7873 | -16.9047 | 841 |
| **7** | Pico das Pedras | 32.7841 | -16.9055 | 883 |
| **8** | Achada do Teixeira | 32.7733 | -16.9081 | 1211 |
| **9** | Achada do Teixeira | 32.7762 | -16.9022 | 1103 |
| **10** | Ribeiro Frio – Viveiro | 32.7354 | -16.8864 | 906 |
| **11** | Ribeiro Frio – Cottages | 32.7319 | -16.8861 | 994 |
| **12** | Chão da Ribeira | 32.7933 | -17.1122 | 519 |
| **13** | Chão dos Louros | 32.7636 | -17.0190 | 748 |
| **14** | Encumeada | 32.7558 | -17.0143 | 999 |
| **15** | Ribeiro Bonito – Levada | 32.8047 | -16.9346 | 568 |
| **16** | Ribeiro Bonito - Ribeiro | 32.7985 | -16.9360 | 560 |
| **17** | Fanal (Lvda. Cedros) | 32.8259 | -17.1580 | 820 |
| **18** | Fanal | 32.8226 | -17.1539 | 889 |
| **19** | Fanal | 32.8182 | -17.1521 | 1023 |
| **20** | Fanal | 32.8062 | -17.1409 | 1134 |
| **21** | Ginjas | 32.7758 | -17.0534 | 869 |
| **22** | Caramujo | 32.7722 | -17.0529 | 981 |
| **23** | Rabaças | 32.7413 | -17.0783 | 993 |
| **24** | Rabaçal | 32.7647 | -17.1341 | 930 |
| **25** | Risco | 32.7608 | -17.1256 | 1048 |
| **26** | Casa do Elias | 32.8268 | -17.1883 | 814 |
